# Endovascular Treatment of Extracranial Internal Carotid Pseudoaneurysm: Description of Three Cases

**DOI:** 10.1055/s-0036-1584168

**Published:** 2016-05-16

**Authors:** Andrea Giorgianni, Carlo Pellegrino, Camilla Micieli, Anna Mercuri, Renzo Minotto, Fabio Baruzzi, Luca Valvassori

**Affiliations:** 1 Department of Neuroradiology, Circolo Varese Hospital, Varese, Italy; 2 Department of Radiology, Circolo Varese Hospital, Varese, Italy; 3 Department of Neuroradiology, Niguarda Ca' Granda Milano Hospital, Milano, Italy

**Keywords:** pseudoaneurysm, internal carotid artery, endovascular therapy

## Abstract

The aim of this study is to explore the possibility of endovascular treatment of internal carotid artery pseudoaneurysm (PSA). These lesions are difficult to treat with a surgical approach, especially if they are located extracranially and close to the skull base. Endovascular stent placement in symptomatic and unstable extracranial internal carotid PSA was found to be safe and effective. Depending on hemodynamic aspects, complete local exclusion of aneurysmal formation is achieved in few months. We present three patients with carotid dissection and PSA formation that have been successfully treated by stent placement.

## Case Reports

### Case One

A 48-year-old man presented at our hospital with left-sided hemiplegia. Computed tomography (CT) showed only right middle cerebral artery (MCA) hyperdensity and indirect sign of thrombosis. Digital subtraction angiography (DSA) showed dissection and complete occlusion of the proximal extracranial tract of the right internal carotid artery (ICA) and a lack of flow in the ipsilateral MCA. After mechanical and fibrinolytic intra-arterial treatment, adequate flow in the MCA and in the ipsilateral carotid was achieved. After 6 months, CT angiography (CTA) showed pseudoaneurysm (PSA; 35 mm) at the C1 level of the right ICA; because the lesion was responsible for stroke, the decision to treat was made after the acute phase, and endovascular treatment was performed. A 6F guiding catheter was placed into the right common carotid artery and one overlapping self-expandable stent (7 × 40 mm; Wallstent, Boston Scientific, Marlborough, Massachusetts) was deployed. The PSA was reduced but clearly visible, filling in the arterial phase after the stent placement, and final checks showed a regular flow in the ipsilateral carotid vessel. CTA after 6 months demonstrated the correct placement of the stent and regular patency of the ICA. The patient recovered completely from his stroke and did not experience further ischemic attacks in the following 2 years.

### Case Two

**Fig. 1:**
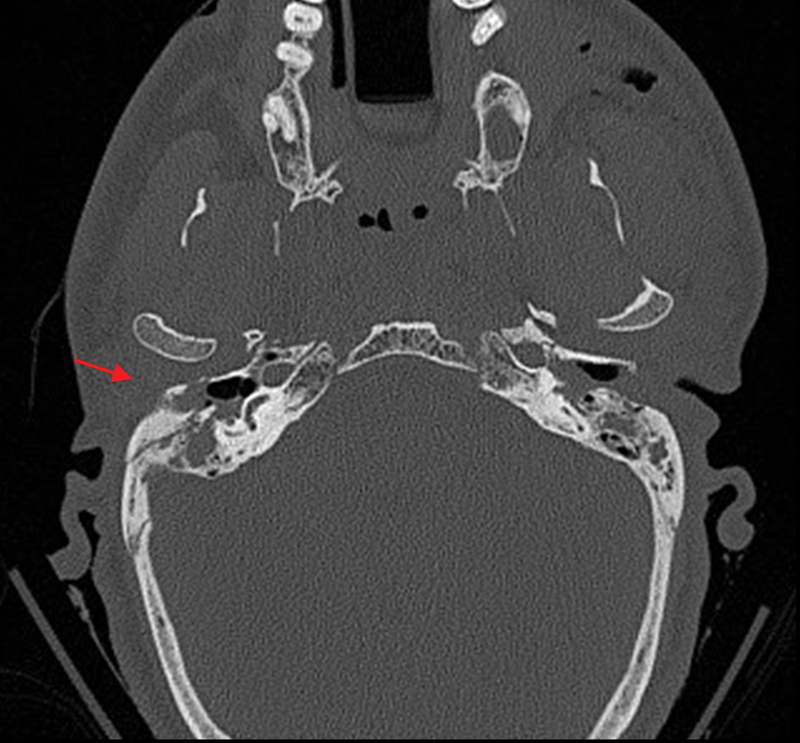
Computed tomography shows mastoid fracture with extension to the external auditory canal (arrow).

**Fig. 2:**
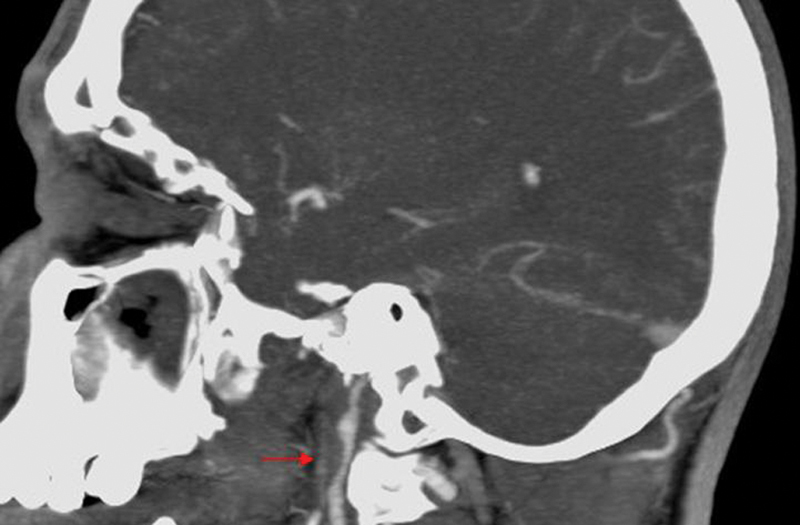
Computed tomography angiography demonstrates the presence of carotid dissection and pseudoaneurysm (arrow).

**Fig. 3:**
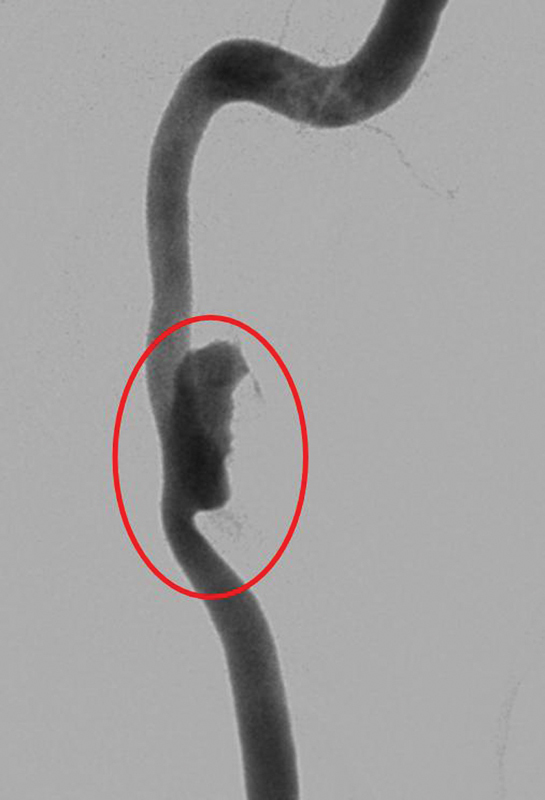
Digital subtraction angiography confirms the internal carotid artery pseudoaneurysm.

**Fig. 4:**
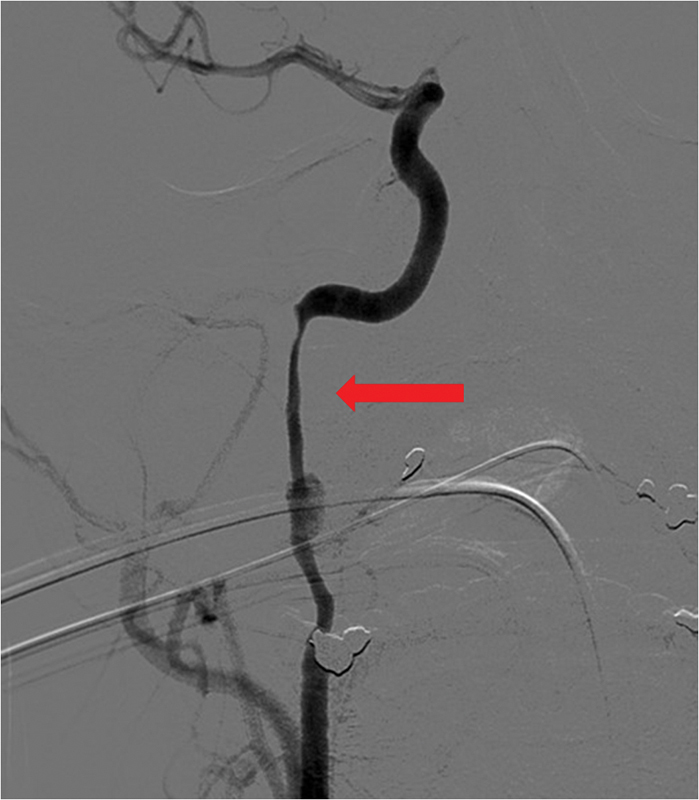
Stent placement procedure. Arrow shows remaining contrast enhancement in the pseudoaneurysm side.

**Fig. 5:**
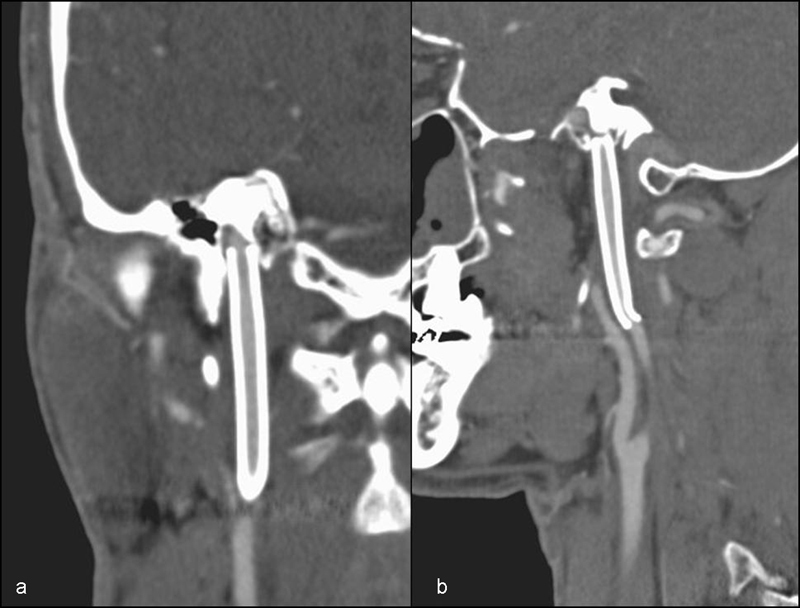
Coronal (a) and sagittal (b) computed tomography angiography scans during follow-up after 6 months shows absence of pseudoaneurysm.

A 47-year-old man presented at our hospital after a vehicular accident. CT revealed severe craniofacial trauma with multiple fractures and scattered hemorrhages ( Fig. 1 ). CTA showed an irregular caliber of the right ICA in the extracranial and intrapetrous tract, with dissection meaning, and regular flow after this level ( Fig. 2 ). During the follow-up (CTA 6 months after the trauma), the PSA has increased in size and its morphology had changed. A new DSA was performed, which confirmed an increase in size of the PSA (8 mm), and for this reason endovascular treatment was performed ( Fig. 3 ). A 6F guiding catheter was placed into the right common carotid artery and an overlapping self-expandable stent (7 × 40 mm, Wallstent) was deployed ( Figs. 4 , 5 ). The PSA was reduced but clearly visible, filling in the arterial phase after the stent placement, and final checks showed regular flow in the ipsilateral carotid vessel. During the 2-year follow-up, there were no further morphologic changes of the PSA.

### Case Three

A 46-year-old woman arrived in our emergency room following a transient ischemic attack (TIA), characterized by numbness of the right upper limb. The CTA revealed a dysplastic appearance of the left extracranial ICA, with a dilated appearance in the first tract and with reduced caliber until the intrapetrous tract, where regular caliber resumed. The DSA confirmed the PSA in the distal extracranial ICA (12 mm). For this reason, heparin was administered for a month in anticipation of endovascular exclusion of the PSA. A month later, an overlapping self-expandable stent (7 × 40 mm; carotid Wallstent) was placed because the injury was symptomatic for TIA. The angiography showed proper stent placement and complete restoration of the carotid lumen. Subsequent CTA checks confirmed the correct exclusion of the PSA, as well as during 5-year follow-up.

## Discussion

The posttraumatic or spontaneous ICA PSAs generally have a benign course when treated with anticoagulant therapy. As previously described by Guillon et al, 65% of aneurysms of carotid dissection do not vary in shape and size, 30% decrease in volume, and only 5% resolve completely. 1 However, in some cases, the natural history of these lesions is burdened with complications such as thromboembolic events that require an invasive treatment. 2 The PSA tends to grow, reducing the true lumen and affecting distal perfusion. For this reason, the safest treatment is required. The surgical approach is very often not feasible to hold the PSA adjacent to the skull base, and the endovascular approach is the only way forward. The purpose of endovascular treatment is to restore the normal anatomy of the vessel, ensuring the patency of the vessel itself. 3 The first endovascular treatment proposed by Lempert et al has been to use metal coils to fill the defect of wall 4 ; this technique has complications such injury to the vessel, and therefore other solutions are preferred. 3 Metal stents are a valid treatment alternative. 5

In the literature, several cases of treatment with uncovered stents and covered polythetrafluorethylene stents are described, but their limited flexibility is the main technical limitation. 6 We treated three patients with extracranial ICA PSAs with carotid Wallstents to optimize lesion thrombosis and to reconstruct the extracranial ICA. 7 In all cases, the lesions were symptomatic or unstable: in the first case, it was responsible for stroke; in the second case, the morphology and the dimensions changed in a short amount of time (6 months); and in the third case, the lesion was symptomatic for TIA. Placing a stent was chosen due to two basic reasons: first, the wide morphology of the collar made it preferable to use the stent instead of the coil; second, the stent allowed the reconstruction of the wall. The placement of an overlapping stent across the aneurysm orifice is relatively simple and safe to decrease the flow inside the PSA. 8 This result is made possible by the porosity of the stent, which increases the turbulence in the PSA, resulting in increased coagulability at that level. This endovascular approach treats not only the injury, but also the vessel from which the damage arises. Among the advantages of stents is the possibility to ensure the patency of the treated vessel and the correct exclusion of the lesion. Although the disadvantages reported in the literature are different, and include no immediate exclusion of PSA, chronic use of antiplatelet therapy, and the clinical and instrumental follow-up, the use of these devices has been shown to be safe and effective. All patients were subjected to dual antiplatelet treatment with Clopidogrel (Plavix, Sanofi Clir SNC, Paris, France) and Cardioaspirin (Bayer, Milan, Italy) 5 days before the treatment and for the following month. A month after the stent placement, Cardioaspirin therapy was continued for 3 months.

This article demonstrates that endovascular treatment is a safe and effective option for the treatment of PSA lesions of the extracranial internal carotid.
